# Unveiling New Horizons: Advancing Technologies in Cosmeceuticals for Anti-Aging Solutions

**DOI:** 10.3390/molecules29204890

**Published:** 2024-10-15

**Authors:** Patrícia Lius Melo Alves, Vitor Nieri, Fernanda de Campos Moreli, Ederson Constantino, Jocimar de Souza, Yoko Oshima-Franco, Denise Grotto

**Affiliations:** Department of Pharmacy, University of Sorocaba (UNISO), Sorocaba 18023-000, Brazil; patilius@gmail.com (P.L.M.A.); nierivitor@gmail.com (V.N.); fernandacamposmoreli@gmail.com (F.d.C.M.); ederson.constantino@prof.uniso.br (E.C.); jocimar.souza@uniso.br (J.d.S.)

**Keywords:** polymers, nanotechnology, anti-aging, cosmeceuticals, delivery system

## Abstract

In the last years, the landscape of anti-aging cosmetics has been marked by significant advances in cosmeceutical delivery systems. This study aimed to conduct a systematic review of these technological innovations, with a focus on anti-aging effects, from 2018 to 2023. The methodology included a thorough search on PubMed and through gray literature, applying rigorous exclusion criteria. The descriptors were selected based on the Medical Subject Headings (MeSH). A total of 265 articles were found. Exclusion criteria were applied, and 90 of them were selected for full reading. After reading the full 90 articles, 52 were excluded, leaving 38 articles for final evaluation composing this review. The key findings highlighted a clear prevalence of studies exploring nanotechnology, including nanoparticles, niosomes, and liposomes. Most of the formulations analyzed in this review emphasize antioxidant activities, which play a crucial role in preventing premature aging caused by free radicals. The reviewed studies revealed specific activities, such as the reduction in melanin synthesis, the inhibition of enzymes involved in the skin aging process, and the prevention of morphological changes typical of aging.

## 1. Introduction

In recent years, the continuous improvement in living standards, along with environmental degradation, has sparked growing interest in methods to prevent or delay skin aging, particularly photoaging. In 2017, the global cosmetics market reached USD 532.43 billion, and is projected to reach 805.61 billion by 2023, demonstrating a compound annual growth rate (CAGR) of 7.14% from 2018 to 2023 [1].

The cosmetics industry uses the term ‘cosmeceutical’ to describe products that claim to impact skin health or have a lasting effect on skin appearance [2]. These formulations are described by the industry as containing therapeutically active ingredients, specifically developed to have reparative effects when applied topically, in addition to their traditional cosmetic use. According to industry claims, these formulations imply a physiological or pharmacological action. Cosmeceuticals are known by various names, such as dermatocosmetics, functional cosmetics, bioactives, and dermocosmetics, among others [3]. Cosmeceuticals serve as a bridge between pharmaceutical preparations and specialized personal care products [4].

Common age-related disorders include wrinkles, reduced skin elasticity, and hyperpigmentation. Wrinkles are a prevalent indicator of human skin aging, resulting from decreased production of collagen and elastin—vital proteins for skin strength and resilience [5]. The decline in skin elasticity with age is largely attributed to the reduction in the production of these proteins. As collagen levels decrease, the skin becomes more susceptible to wrinkles, sagging, and loss of a youthful appearance. This process is further accelerated by external factors such as sun exposure and pollution, which generate reactive oxygen species (ROS) that degrade collagen and impair its production. Additionally, hormonal changes, especially during menopause, exacerbate this reduction in skin elasticity [6].

Currently, the perception of skin health and beauty is associated with overall well-being, leading to a growing demand for anti-aging products. Although no topical treatment or ingredient has been shown to completely halt photoaging, literature reviews suggest that some products may reduce and delay the signs associated with this process. Ingredients such as retinoids, antioxidants, and sunscreens have been identified in research as effective in mitigating the effects of photoaging and promoting skin health [7].

In the current landscape, the demand for cosmetics that offer deeper and longer-lasting results is strongly driven by innovations. There is an increasing emphasis on investigating the stability and enhanced efficacy of active ingredients [8]. Significant advances in drug delivery systems have been the subject of research, including the development of new encapsulation strategies to effectively protect and transport active ingredients. Researchers have also focused on manipulating the release timing of the active principle, which refers to controlling how quickly the active ingredient is released at the target area. Additionally, targeted delivery techniques are being explored to direct drugs specifically to the intended site of action, enhancing efficacy and reducing side effects [9].

The efficacy of a cosmetic depends not only on the active ingredients but also on the technology used in its formulation. The primary function of the vehicle is to efficiently deliver the active ingredients to the desired location and ensure that they remain there long enough to produce the intended effect [10]. In this context, the use of nanoparticles has been widely explored by the cosmetics industry. Nanoparticles are small particles with dimensions measured in nanometers, typically ranging from 1 to 100 nanometers. Due to their reduced size, they exhibit unique physical, chemical, and biological properties compared to bulk materials, making them useful in various fields, including medicine, cosmetics, and electronics [11].

Nanotechnology aims to enhance various characteristics such as durability, water resistance, strength, and conductivity. Nanomaterials are increasingly being selected for their ability to overcome common limitations in cosmetics, such as penetration, stability, and controlled release of active ingredients [12].

The development of these nanotechnology-based products includes a variety of nanomaterials with distinct compositions, sizes, and shapes, with nanocarriers being one of the most common examples. The essential function of nanocarriers is to transport and deliver bioactive agents to target tissues; they can be made from various materials with different structures. Their primary characteristic is particle size, which plays a crucial role, as it directly influences the biological properties of the carrier [13]. Complementing this approach, nanoemulsions emerge as a promising example of nanocarriers, offering advantages such as transparency, low viscosity, and high efficiency in penetrating active ingredients—characteristics that make them ideal for cosmetic applications [14].

In this context, nanotechnology has found applications in the field of cosmetics, giving rise to innovative formulations known as nano-cosmeceuticals. These include nanoformulated cosmetics using polymer-based nanostructured systems (such as nanocapsules and nanosheres), lipid-based nanostructured systems (such as liposomes, niosomes, and nanoemulsions), and metal-based nanostructured systems (such as gold and silver nanoparticles), among others. Each of these has specific characteristic properties that enhance drug delivery, increase absorption, potentiate cosmetic efficacy, and offer many other benefits [8,15].

Although the unique properties of nanomaterials make them desirable for cosmetic applications, as they can perform specific functions, they may also pose health risks to consumers. The potential health risks associated with insoluble nanoparticles are a widely debated topic in the scientific literature, primarily due to conflicting results and the lack of long-term toxicological studies [16]. Therefore, to determine whether a nanomaterial is safe for human health and the environment, a range of parameters must be assessed, with particular emphasis on advanced physicochemical characterizations [17].

There is still no global consensus on the definition of nanomaterials as cosmetic ingredients. As a result, each country adopts its own definition and regulation. The United States and the European Union are the two largest cosmetic markets [18]. The EU, in its Regulation EC 1223/2009, defines nanomaterial as ‘an insoluble or biopersistent material intentionally manufactured with one or more external dimensions, or an internal structure, in the size range of 1 to 100 nm’. In the United States, the FDA (Food and Drug Administration) has yet to adopt an official definition for nanomaterials. However, in 2014, the agency highlighted two criteria for assessing whether a regulated product involves the application of nanotechnology: (1) whether ‘a material or final product is designed to have at least one external dimension, or an internal or surface structure, in the nanoscale range (approximately 1 nm to 100 nm)’; and (2) whether ‘a material or final product is designed to exhibit properties or phenomena, including physical or chemical properties or biological effects, attributable to its size, even if those dimensions exceed the nanoscale, up to one micrometer (1000 nm)’ [19].

Understanding these delivery systems with potential anti-aging effects is necessary to elucidate the latest cosmeceutical technologies aimed at delaying or minimizing the risk of skin aging. The objective of this study was to systematically review the technological advancements in cosmeceutical delivery systems over the past five years, focusing on anti-aging effects.

## 2. Results

### Review Methodology: Article Selection and Analysis

**Figure 1:**
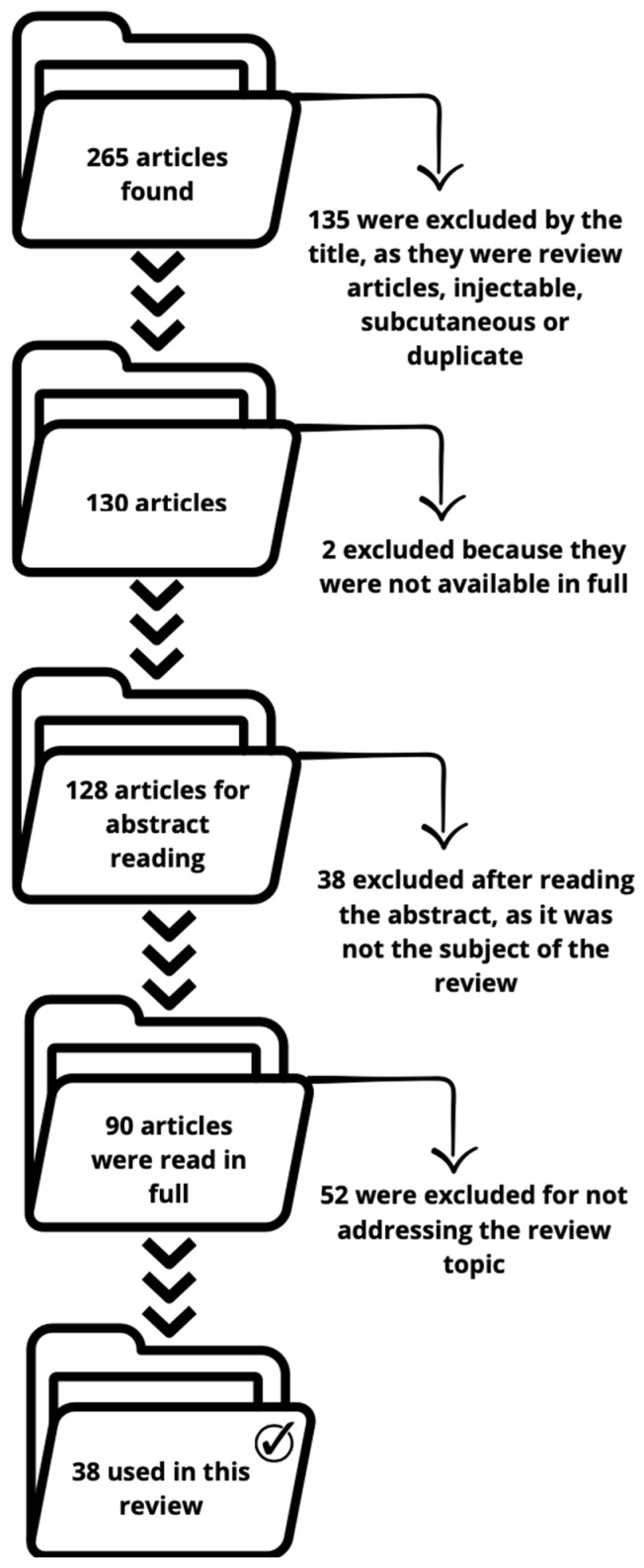
Establishment of exclusion criteria and the leaving articles.

For this review, only articles related to delivery systems focused on anti-aging cosmetics for topical use were considered. Figure 1 illustrates the search process carried out in the PubMed database and gray literature, in which 265 articles were identified. Of these, 135 were excluded after reading the titles, as they were either reviews or indicated in the title that the study addressed injectable or subcutaneous substances. After this initial exclusion stage, 130 articles remained, 2 of which were discarded for not being freely available in full, resulting in 128 articles for the next screening phase. Of the 128 articles, 38 were excluded after reading the abstracts, as they did not fit the theme of the review. The remaining 90 articles were read in full, and from this total, 52 were eliminated for not focusing on anti-aging solutions. Thus, 38 articles were included in the final evaluation that supports this review.

After the completion of the final article selection, the results were organized into three tables to provide a clearer visualization of the outcomes. In Table 1, there are in vitro and ex vivo studies; in Table 2, there are in vivo studies (conducted on animals); and in Table 3, there are human studies (clinical trials). Only two studies addressed ex vivo were grouped in the same table as in vitro studies. Each table presents the main outcome of the study, the delivery system used, the active compound, along with the corresponding reference.

Table 1 presents the results of the selected in vitro studies, which involve experiments conducted outside living organisms, generally microorganisms, cells, or molecules extracted from their normal biological context. The use of in vitro techniques is essential for the advancement of scientific knowledge, providing a solid experimental foundation for the interpretation and application of results obtained in more complex biological models.

The selection of articles for in vitro analysis comprised 17 publications that met the predefined criteria. Among these, a prevalence was observed in articles featuring delivery systems based on nanoparticles, followed by those employing niosomes and liposomes as release systems. In only one article found, ethosomes and transetosomes were used as a release system, which have similar characteristics to liposomes and niosomes.

Table 2 presents the results of in vivo studies, with the majority of rats and mice being used to carry out skin contact or compound diffusion tests. In vivo studies refer to experimental protocols using animals, conducted after approval of the research by a respective ethics committee. In this review, analyzes were carried out through the topical application of the developed formulations. Articles that met the predefined inclusion criteria totaled 10, including articles covering in vitro and animal tests. The predominance of nanotechnology through the delivery of nanoparticle assets is observed in eight studies. Additionally, there are two studies that use micelles and emulgel as their delivery modes. The observed results present confirmed anti-aging actions and a positive result on each delivery system.

Table 3 presents the results of the studies in humans. Human studies refer to clinical trials conducted in a selected population, after research approval by an ethics committee. This type of research corresponds to the final phase of testing for the delivery of new products. In this review paper, clinical trials were conducted through the topical application of the developed formulations. The papers that met the predefined inclusion criteria totaled 10, including papers encompassing in vitro, animal, and human trials described only in this table. The predominance of nanotechnology through the delivery of nanoparticulate actives is observed with five articles, followed by two microparticulate actives. Additionally, there are three articles in which the particle size is unknown as the article does not mention it. The results obtained through the formulations indicate anti-aging action in all studies selected in this review, with anti-wrinkle action and skin hydration being the most observed results.

Table 4 presents the cosmeceutical delivery systems found in this review, with the main ones being lipid-based nanosystems—liposomes, niosomes, solid lipid nanoparticles (SLNs), nanoemulsions—and metal-based nanosystems—metallic nanoparticles. The delivery systems differ in physical and chemical properties, but a significant trend of utilizing nanotechnology is noticeable in the majority of the selected studies. It has become evident that the use of nanotechnology in cosmetics is becoming a crucial tool for scientific investigation as well as the development of new cosmetic products. It was remarkable to observe that, in cosmetic formulations, a considerable amount of nanoscale materials is already being introduced or recommended for future use, both as nanosystems and as new nanocarriers designed to encapsulate active ingredients. This aims to facilitate their effective delivery through the skin barriers.

In the in vitro studies analyzed in Table 5, significant gaps were identified regarding the limitations of the work, particularly concerning the long-term effects and practical applicability of these technologies. For example, some studies demonstrated the administration of compounds through delivery systems based on niosomes, highlighting their anti-aging activity but failing to delve into the mechanisms of action of the active principles [20]. Similarly, another article explored the immediate effects of pre-treatment against UV stress without considering the cumulative impacts over time, which are essential for understanding the total effect on skin aging [21]. The lack of data on long-term efficacy emerged as a recurring theme. In one study, the incubation effects were limited to short periods (48 to 72 h), with no evaluations that considered prolonged impacts [23]. In another study, funded by Inoliva Ilac ve Gida A.S., the authors did not present data assessing the long-term anti-aging effects of the formulations and did not discuss potential conflicts of interest, a crucial aspect for ensuring research transparency [22]. Furthermore, limitations were observed in quantification methods [24] and in the measurement of a restricted number of inflammatory response markers [25]. The stability of some delivery systems also raised questions, as they require stringent storage conditions to maintain their properties [26]. This concern is compounded by the inherent instability and easy degradation of certain compounds, such as ascorbic acid [28]. Additionally, the studies lack data on adverse environmental conditions, such as light exposure and humidity, which represent a significant obstacle to the practical application of the formulations [34,36]. The absence of long-term safety tests [29,32,33] and results from delivery systems that did not provide information on skin penetration and the lasting effects of topical application were also noted [31]. In summary, although the reviewed studies demonstrate promising advancements in the use of innovative delivery systems in cosmeceuticals, most still need to investigate long-term effects and the practical applicability of these formulations in the context of skin aging and other cosmetic applications.

The analysis of the limitations found in in vivo studies (Table 6) revealed several gaps that may impact the interpretation and applicability of the results. Several works [37,38,42,43,44] exhibit deficiencies in critical information, such as the age of the animals used and the number of individuals tested [38]. The absence of details regarding age may compromise the validity of the results, as the developmental stage of the animals is fundamental in evaluating biological responses. The lack of robust stability tests is a recurring limitation. Studies 38 and 47 did not provide information on the conduct of stability tests, while study 41 did not mention long-term tests under varying temperature and humidity conditions. Furthermore, study 39 highlighted the physical instability of emulsions containing L-ascorbic acid, suggesting that the formulation may not be reliable. This gap in methodology could undermine the effectiveness of the formulations in the long term. In studies 40 and 45, adult rats aged between six and eight weeks were used; however, the methodology did not clarify whether these animals were in an appropriate phase for anti-aging investigations. The lack of clarity regarding the conditions of induced aging in the animals limits the applicability of the findings in broader contexts. Study 47 revealed size-dependent toxicity of nanoparticles but did not explore the underlying mechanisms of this phenomenon. In summary, the identified limitations, including the absence of information on the age of the animals, the lack of stability tests, and insufficient characterization of the mechanisms of action, indicate the need for a more rigorous and detailed approach in future investigations.

The clinical studies presented in Table 7 analyzed the efficacy of anti-aging products, revealing several limitations. Some studies included potentially harmful ingredients in the tested formulations, such as parabens, which may impact the safety and acceptability of the products [48,52,57]. Furthermore, many studies had a limited number of participants; for example, one study had only 13 healthy Asian women aged between 25 and 40, which may not adequately represent the efficacy of the product in older age groups—a crucial factor for anti-aging products [49]. Another point to consider is that some studies were conducted using a single-blind methodology [49,50] and in single centers [49,50,52], further compromising the robustness of the results. In contrast, the presence of conditions like melasma among the participants was considered an advantage in assessing the treatment’s efficacy [50]. In one study, the sample consisted of only six healthy volunteers, all with normal skin, which compromised the generalization of the results due to the small and unrepresentative sample size [51]. Moreover, another study focused on specific age groups, such as the anti-aging mask tested on individuals aged 42 to 64 and the purifying mask on participants aged 25 to 40, allowing for a more targeted evaluation; however, the small sample size still limited generalization [56]. Finally, the research involving healthy men also had limitations, with only 13 participants, and the conclusion was generic, highlighting promising effects without providing specific evidence regarding the efficacy of the tested products [57]. Overall, the main limitations identified were the small number of volunteers, inadequate or unreported age, and lack of information about the quality of the volunteers’ skin. Additionally, some studies provided generic conclusions, emphasizing promising anti-aging effects from topical application without delivering specific evidence of the efficacy of the tested product [52,53,57]. Only in one of the studies, compared with the others, no significant limitations were observed [55].

## 3. Discussion

Cosmetics are an indispensable element of daily routines. Moreover, the incorporation of nanotechnology into cosmetics has heightened its widespread acceptance among users globally. As evidenced by the presented outcomes, innovative nanocarriers such as liposomes, nanoemulsions, niosomes, lipid nanoparticles, polymeric nanoparticles, microparticles, etc., are employed in the formulation of diverse cosmetics and cosmeceuticals, leading to enhanced results. The review highlights the prevalence of nanoformulations, indicating a notable advantage in integrating nanocomponents into cosmetic products. This advantage includes the improvement of stability for inherently unstable cosmetic ingredients in formulations, increased permeability of specific active ingredients, improved effectiveness and tolerance of UV absorbers, and an overall enhancement in aesthetic appeal of the products [3]. According to Bilal et al. [1], these nano systems can assist in the absorption or penetration of active portions of cosmeceuticals into the epidermal layers in a remarkable way, achieving either a burst or sustained release of active ingredients.

The delivery systems differ in physical and chemical properties, and the main ones found in this review are liposomes, niosomes, solid lipid nanoparticles (SLNs), nanoemulsions, and metallic nanoparticles. Liposomes (LP) are biodegradable vesicles consisting of a spherical phospholipid bilayer enclosing an aqueous compartment. The most commonly used lipids in LP preparations include phospholipids, such as phosphatidylcholine, phosphatidylethanolamine, sphingolipids, glycolipids, and sterols. LP come in diameters ranging from 20 nm up to a few hundred micrometers [58]. They can be categorized as either unilamellar or multilamellar vesicles. Both hydrophilic and lipophilic ingredients can be encapsulated into LP within the polar and oil-soluble cavities, respectively [59].

Niosomes are vesicles composed of non-ionic surfactants—amphipathic compounds with an overall neutral charge. These structures are analogous to phospholipid vesicles, such as LP, demonstrating the capability to encapsulate aqueous solutes and function as carriers for drugs. The cost-effectiveness, enhanced stability, and consequent ease of storage associated with non-ionic surfactants have prompted their utilization as viable substitutes for phospholipids [60]. Solid lipid nanoparticles are considered colloidal delivery systems and represent innovative delivery systems consisting of a monolayer shell encapsulating a lipid core and finding application in the formulation of cosmeceuticals. These formulations feature a solid-state lipid matrix with a nanometer size [61].

Nanoemulsions are liquid dispersions considered to be kinetically or thermodynamically stable, where an oil phase and an aqueous phase are combined with a surfactant. Depending on the composition, nanoemulsions can be categorized as oil-in-water, water-in-oil, or bicontinuous. The particle sizes in these nanoemulsions range from 50 to 200 nm. These dispersed phases contain small particles or droplets with very low oil/water interfacial tension. Nanoemulsions have a lipophilic core surrounded by a monomolecular layer of phospholipids, making them particularly suitable for delivering lipophilic compounds [62].

Among the metallic nanoparticles, gold, silver, zinc oxide, and titanium oxide are highlighted. Gold nanoparticles have gained prominence in cosmetic formulations due to their effectiveness against bacterial and fungal infections. These particles range in diameter from 1 to 100 nm. Various biological activities are achieved through the use of gold nanoparticles, including rubefacient effects, enhancement of skin metabolism, and the slowing down of aging symptoms [63]. Similar to gold nanoparticles, silver nanoparticles also exhibit remarkable activity against bacteria and fungi. Formulating these nanoparticles into skin ointments provides additional benefits as a disinfectant, and they are also used in oral cosmeceuticals [64].

Zinc oxide (ZnO) nanoparticles are widely used in cosmetics, sun care products, coatings, and antibacterial products. In addition to their ability to block UVA and UVB rays, these nanoparticles are incorporated into various cosmetic formulations to protect against the long-term harmful effects of these rays, such as skin aging and skin cancer [65]. Titanium dioxide (TiO_2_) nanoparticles are included in various cosmetic products as UV filters. Titanium dioxide primarily functions by reflecting UVB radiation. Thus, the combined use of TiO_2_ and ZnO nanoparticles provides excellent protection against solar radiation without causing the skin irritation often associated with chemical UV filters [66].

In our investigation, we noted a wide array of active ingredients, emphasizing the diverse approaches employed in the development of anti-aging products. Each component brings unique benefits, contributing to a more comprehensive strategy in alleviating skin tissue damage. The literature reviewed reveals a diversity of natural ingredients, including gallic acid, *Myrtus communis* extract, glutathione tripeptide, *Ocimum sanctum* extract, lycopene, royal jelly, epigallocatechin gallate (EGCG), resveratrol, myricetin, ascorbic acid (AA), nicotinamide (NIC), morin, bakuchiol, and curcumin. These extracts generally offer therapeutic benefits or drug-like properties capable of influencing the biological functions of the skin based on their constituents [20,27,28,50,51]. They represent comprehensive skincare solutions that go beyond mere aesthetic improvement, contributing to enhanced skin function and appearance. These ingredients stimulate collagen production, combat the harmful effects of free radicals, maintain keratin integrity under challenging conditions, and promote healthier skin.

A majority of the formulations identified in this review highlight antioxidant activities, crucial in preventing premature aging induced by free radicals. Oxidative stress is considered a significant mechanism in skin aging, triggering the apoptosis of many cell types, especially human skin fibroblast cells, crucial for maintaining youthful and healthy skin [5,7,32,36,43]. The ability to neutralize free radicals is essential to protect the skin against oxidative damage. The specific activities identified in the studies of this review, such as melanin reduction, inhibition of enzymes related to skin aging, and prevention of morphological changes associated with aging, point to promising results.

These targeted actions contribute to a more holistic approach to reducing the signs of aging. Features such as biocompatibility, non-cytotoxicity, and long-term stability emphasize the importance of developing products that are safe for topical use. These attributes are essential for ensuring the acceptance and effectiveness of anti-aging products. Although the results are promising, it is important to recognize potential challenges, such as the stability of certain ingredients.

Most of the formulations highlighted in the studies demonstrated effectiveness in addressing signs of aging, ranging from wrinkle reduction to increased hydration, enhanced elasticity, and diminished spots. Several articles highlight that their formulations outperform conventional treatments, such as chemical peels, indicating a potential breakthrough in the field. The observation of clinical and visual improvements, such as the recovery of skin damaged by UV radiation and visible anti-wrinkle effects, strengthens the practical applicability of these formulations. The studies demonstrate improvements in the skin’s ultrastructure and suggest that some formulations have effects beyond the surface, potentially influencing deeper cellular processes.

According to the findings, in in vitro studies, the efficacy of various formulations is highlighted, including niosomes with gallic acid, NanoPCL-M nanofibers with natural extract of *Myrtus communis*, propolis polyphenols delivery systems, NLC with *O. sanctum* extract, and systems containing lycopene, morin, GA–AuNPs, among others. These formulations demonstrate antioxidant activities, inhibition of enzymes related to aging, protection against oxidative stress, and other beneficial effects. Human studies assessed specific formulations, including those that provide anti-aging action, recovery of the skin from imperfections related to sun exposure, effective treatment for melasma, visible improvements in wrinkles and skin texture, and increased skin hydration, elasticity, and smoothness.

Certain ingredients, such as gallic acid, glutathione tripeptide, *O. sanctum* extract, EGCG, resveratrol, myricetin, morin, CA (telomerase activator), GA–AuNPs, bakuchiol, curcumin, propolis, adenosine, niacinamide, and sulforaphane, are featured in both in vitro and in vivo tables, indicating anti-aging activities, such as wrinkle reduction, improvements in skin hydration and elasticity, and protection against oxidative stress. This suggests a consistent exploration of promising anti-aging compounds from the laboratory settings to human trials. The correlation between in vitro and human studies suggests a positive transition from laboratory results to clinically observable benefits.

Delivery systems, such as niosomes, nanofibers, lipids, nanoemulsions, and nanocomposites, have proven effective in both scenarios, emphasizing the importance of suitable delivery systems to optimize the absorption and efficacy of active ingredients. Some formulations, such as the antioxidant efficacy of GA–AuNPs and the reduction in melanin by SLNs containing EGCG, resveratrol, and myricetin, can be directly compared.

Additional findings reveal that some formulations showed visible improvements in the short term, while others, such as “anti-aging masks” and “lifting” treatments, demonstrated longer-term benefits, implying different treatment durations for specific outcomes. However, it is important to emphasize that these observations provide an overview of the progression from in vitro to clinical studies. Careful consideration of experimental conditions, as well as the limitations and inherent variabilities in each system is essential for a comprehensive understanding.

Nanomaterials such as liposomes, nanoparticles, and nanoemulsions can enhance the penetration of active ingredients into the skin, allowing for more controlled and targeted release. This results in greater bioavailability, improving efficacy with lower doses. While conventional formulations are widely effective, particularly in anti-aging treatments and acne management, they may encounter absorption limitations, with effects concentrated in the more superficial layers of the skin [67].

The cosmetics industry claims that nanotechnology can minimize adverse effects by enabling the controlled release of actives in smaller doses. However, there are concerns regarding the long-term release of nanoparticles, which may penetrate biological barriers and enter systemic circulation. In contrast, conventional substances such as retinoids are known to cause irritation, redness, and peeling, especially in sensitive skin, although their long-term safety is well documented [68].

Cosmetic products based on nanotechnology tend to be more expensive due to advanced manufacturing processes and the research required to develop safe and effective formulations. This can raise the final price for consumers, whereas conventional formulations are widely available and more affordable due to large-scale production and greater standardization of use [69].

Conventional formulations, particularly retinoic acid, are highly unstable when exposed to air and light, which can reduce their efficacy over time if not stored properly. On the other hand, nanoparticles can enhance the stability of active ingredients that normally degrade quickly when exposed to air or light, prolonging the shelf life of products [70].

Due to their sustained release capability of active ingredients into the skin, nanotechnology products allow the actives to act for extended periods, reducing the need for frequent reapplication. In contrast, conventional products generally have a more immediate and superficial release, which can lead to localized concentration peaks, increasing the risk of irritation [71].

This review noted that the cosmetics industry claims nanotechnology provides significant advancements, particularly in efficacy, precise release, and safety with lower doses. However, concerns about its long-term impact and higher costs remain. In contrast, conventional products continue to be a reliable choice due to their affordability and well-established safety profile, despite potential side effects.

The data analysis reveals that, although the evaluated studies offer promising advances in the application of innovative delivery systems in cosmeceuticals, various methodological and long-term limitations compromise the robustness and applicability of the results. In the in vitro studies, there was a lack of investigations into the mechanisms of action of the active ingredients, as well as the absence of evaluations of cumulative long-term effects. These factors are essential for understanding the long-lasting efficacy of anti-aging cosmeceuticals, as skin aging is a continuous and multifactorial process. Additionally, the lack of data on formulation stability and the impact of environmental conditions, such as light and humidity, raises questions about the practical viability of these technologies for end consumers. The stability of compounds like ascorbic acid, known for its easy degradation, is a crucial example that highlights the need for greater efforts to ensure the efficacy of products over time.

In vivo and clinical studies also present limitations such as small sample sizes, lack of age diversity among participants, and the absence of long-term stability tests, all of which emerge as significant issues. The presence of potentially harmful ingredients in some tested formulations, such as parabens, and the use of simple methodologies, such as single-blind studies conducted in single centers, reduce the reliability of the results. The lack of clarity regarding the conditions of induced aging in animals and insufficient information on size-dependent nanoparticle toxicity further indicate that greater rigor is needed in future investigations. In summary, while innovative delivery systems show great potential, there is a clear need for more comprehensive studies that consider long-term effects, formulation stability, and participant profile diversity to ensure effective practical translation.

Finally, we observe that several countries are dedicated to the study and development of new anti-aging cosmetics and active delivery methods. This review highlights significant contributions from researchers in Egypt (six articles), Portugal, Italy, and Iran (with n = three each), Taiwan, Thailand, Pakistan, Greece, and China (with *n* = two each), and Turkey, Poland, Japan, Indonesia, France, Spain, Korea, Canada, and Saudi Arabia, with one article each. This global collaboration of researchers reflects the diversity and international commitment to advancing anti-aging science. These findings indicate a combination of factors that may contribute to a growing interest in skin aging research, such as climate and environmental conditions of intense sun exposure. Climatic characteristics may trigger the need to develop specific solutions to protect and treat the skin, and as awareness of skincare grows, researchers may be responding to this demand by working on the development of more effective products.

In conclusion, the thorough investigation into diverse delivery systems and active ingredients in anti-aging formulations reveals a dynamic landscape in the cosmeceutical field. Innovations in active delivery methods demonstrate promising progress in the quest for effective and safe solutions to combat signs of skin aging. The widespread use of nanotechnology, exemplified by liposomes, nanoparticles, and niosomes, marks a significant advancement. The transition from in vitro studies to human trials indicates a promising continuity, with nanocarriers demonstrating significant anti-aging effects. Improvements in skin hydration, elasticity, and reduction in wrinkles, along with the sustained benefits of long-term treatments, offer a holistic perspective on the potential of these nanoformulations. While challenges in ingredient stability remain, the remarkable progress in addressing oxidative stress and other aging-related factors through nanotechnology suggests a future where cutting-edge cosmeceuticals, leveraging nanocarriers, will play a crucial role in promoting skin health. This review contributes to the ongoing dialogue in the field, emphasizing the importance of targeted, effective, and safe nanotechnology-based anti-aging solutions that meet the evolving demands of skincare.

## 4. Materials and Methods

**Figure 2:**
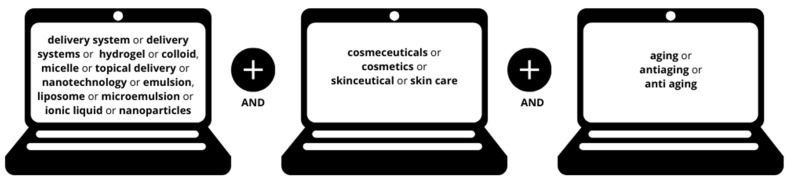
Descriptor combinations overview, based on the ‘Medical Subject Headings (MeSH).

Systematic electronic research was conducted on two search platforms (PubMed and gray literature) on 24 August 2023. The combined descriptors used as search a strategy in the literature are shown in Figure 2. The descriptors were selected based on the ’Medical Subject Headings (MeSH),’ and they were selected to be sufficiently specific, avoiding the inclusion of overly broad or irrelevant literature.

The research was conducted over a five-year period, from 2018 to 2023, and focused solely on the English language. Only original articles related to delivery systems focusing on anti-ageing cosmetics for topical use were considered for this review.

**Figure 3:**
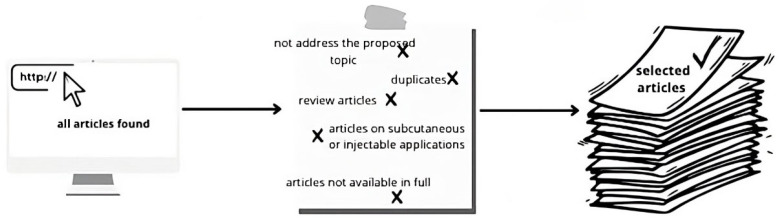
Exclusion criteria selection process leading to analyzed articles.

As shown in Figure 3, exclusion criteria included articles not addressing the proposed topic, duplicates, review articles, articles on subcutaneous or injectable applications, and articles not available in full. The final selection was restricted to works related to delivery systems for cosmetic formulations with anti-aging effects.

To conduct a meticulous article selection, five evaluators participated in all stages of the process. At each subsequent phase, the articles were randomly redistributed among the evaluators to ensure the reliability of the results.

## Figures and Tables

**Table 1:** Summary of data collected from in vitro studies on new cosmeceuticals delivery systems selected by the exclusion criteria mentioned above.

Results	Delivery System	Active Compound	Reference
The cationic CTAB niosome loaded with gallic acid demonstrated anti-skin aging activity, including a melanin suppression effect, antioxidant activity, and the inhibition of matrix metalloproteinase 2.	Niosomes	Gallic acid	[20]
The combination of *Myrtus communis* natural extract and the polycaprolactone nanofibrous scaffold (NanoPCL-M) resulted in a significant reduction in senescent cells, anti-aging effect of the pretreatment, and also prevented morphological changes associated with aging in keratinocyte populations organized in a 3D structure.	Polycaprolactone nanofibrous scaffold	*Myrtus communis* extract	[21]
The results demonstrate a successful anti-aging cosmeceutical preparation containing glutathione tripeptide loaded lipid-based niosome dispersion, suitable for topical use due to its textural and viscosity properties.	Emulgel with niosome	Glutathione tripeptide	[22]
The delivery system can preserve the anti-mutagenic, anti-oxidative, and anti-ageing effects of propolis polyphenols at levels similar and comparable to those of propolis methanolic extracts, making the system ideal for applications requiring non-toxic solvents and controlled release of the polyphenol content.	Liposomes	Propolis polyphenols	[23]
The Nanostructured lipid carriers (NLC) system containing *O. sanctum* extract was an attractive dermal delivery system, ensuring safety and enhancing the dermal delivery of Rosmarinic acid. This could be used in future topical anti-aging products.	Nanostructured lipid carriers, nanoemulsion, liposome, and niosome	*Ocimum sanctum* Linn. extract	[24]
The self-emulsifying system containing Lycopene prevents DNA degradation, exhibits antioxidant activity, and inhibits enzymes (tyrosinase and elastase) involved in the skin aging process, without causing cytotoxicity in HaCaT cells.	Self-emulsifying	Lycopene	[25]
The study addressed the stability issues of royal jelly, maintaining its stability for 6 months. Furthermore, the double encapsulation of royal jelly in cyclodextrin/liposomes allows a time-controlled release of 10-HDA, potentially valuable for dermal applications. The novel delivery system aims not only to eliminate the stability challenges of the sensitive royal jelly component, 10-HDA but also to achieve controlled release, particularly of 10-HDA, enhancing bioactivity in cosmeceutical applications.	Liposomes	Royal jelly (10-hydroxy-2-decenoic acid)	[26]
The combination of EGCG-loaded solid lipid nanoparticles (SLNs) with resveratrol-loaded SLNs proved to have the highest protection against induced oxidative stress. Encapsulating EGCG, resveratrol and myricetin in SLNs seems to be a suitable strategy for the delivery of these antioxidants to the skin, improving their bioavailability	Solid lipid nanoparticles	Epigallocatechin Gallate (EGCG), resveratrol and myricetin	[27]
Microparticles containing AA and NIC showed antibacterial activity, suggesting they may be a useful additive in cosmetic products. Together, these results describe a technology based on a natural polymer for skin cosmetic products, facilitating the topical release of antioxidant drugs.	Encapsulated microparticles (Chitosan)	Ascorbic acid (AA) and nicotinamide (NIC)	[28]
Both morin and liposomal morin effectively protected keratinocytes against ROS generation induced by PM exposure. They further provided anti-aging and anti-skin pollution properties by reducing MMP-1 expression via downregulation of the MAPK signaling pathway.	Liposomes	Morin	[29]
Morin-loaded niosome had potent anti-aging (antioxidative and anti-stain) activities, including reduction in melanin formation due to tyrosinase inhibitory activity and intracellular ROS scavenging activity. The product demonstrated effective UVR protection (SPF > 30).	Niosomes	Morin	[30]
The results suggest that CA could permeate the skin barrier, both when encapsulated in transetosomes and when included in cosmetic creams containing CA-loaded transetosomes. These findings provide opportunities for research and development in other applications of the encapsulation of CA, a telomerase activator, in topical products such as cosmetics, as anti-aging active ingredients, or even in topical pharmaceuticals and medicines.	Liposomes, ethosomes, and transethosomes	Cycloastragenol (CA)	[31]
The GA–AuNPs inhibited oxidative stress, MMP-1 upregulation, and the release of type I collagen by high glucose. Thes study suggests that GA–AuNPs are valuable as an active ingredient in anti-aging products, particularly for glycation-induced skin aging.	Metallic nanoparticles	Gallic acid	[32]
*Hubertia ambavilla* extract contains bioactive constituents used for synthesizing gold nanoparticles with interesting properties, particularly for cosmetic applications. GAuNP are non-toxic to fibroblast and dermal cells, efficiently scavenging free radicals. They also protect against UV-A induced damage to fibroblasts and dermal cells.	Metallic nanoparticles	*Hubertia ambavilla*	[33]
The improved permeability and antiaging potential of the bakuchiol-encapsulated rich extract were observed, indicating that the obtained ecological nanoemulsions are competitive with commercial retinol formulations.	Nanoemulsions	Bakuchiol (bioretinol)	[34]
A curcumin nanoformulation, sized at 77 nm and containing 3% ethanol, proved more effective in increasing β1-integrin gene over-expression, preventing apoptosis in fibroblast cells, and decreasing Bax and NFκB gene expression compared to a particle size of 50 nm. Such a formulation may be considered a valuable candidate in anti-aging and wound-healing formulations.	Nano-carriers	Curcumin	[35]
IB@NPs showed excellent biocompatibility, inhibited oxidative damage to mouse NIH-3T3 fibroblasts, and reduced intracellular ROS generation, as indicated by an in vitro DPPH antioxidant assay. Therefore, as carriers with excellent stability, IB@NPs hold potential applications in cosmetics and pharmaceuticals.	Nanoemulsions	Idebenone (IB)	[36]

**Table 2:** Summary of data collected from in vivo studies on new cosmeceuticals delivery systems selected by the exclusion criteria mentioned above.

Results	Delivery Sistem	Active Compound	Reference
Protransf-CoQ10 emulgel increased collagen density and the number of fibroblast cells in UV radiation-induced skin-aged mice, indicating its potential for repairing the skin aging process. CoQ10 emulgels, including Protransf-CoQ10 emulgel, significantly increased the number of fibroblasts compared to the control group, indicating increased collagen production.	Emulgel	Coenzyme Q10	[37]
Topical application of Nanolipoic acid (nLA) effectively improved UV-induced pigmentation and epidermal thickening, demonstrating the efficacy of nanoencapsulation in enhancing α-lipoic acid’s therapeutic potential.	Micelles	α-lipoic acid	[38]
FucoPol-based cream showed positive physical–chemical, rheological, stability, antioxidant, and bioactivity properties throughout 60 days. The potential of FucoPol for use as a standalone anti-aging cream or for enhancing UV-blocking capacity of other formulations has been established.	Micelles	α-lipoic acid	[39]
Caffeinated hyalurosomes were successfully developed as an anti-aging nanoplatform for caffeine delivery. They exhibited prolonged in vitro drug release and superior in vivo anti-aging efficacy compared to caffeine solution or caffeine gel.	Hialurosomes	Caffeine	[40]
*Citrus sinensis* L. peel standardized extract (CSPE) exhibits potent anti-aging activity via downregulation of MMP1 mRNA expression, anti-inflammatory, and antioxidant effects. The visible appearance of UV-induced photoaging in mice significantly improved after the topical application of CSPE-NLC cream for 5 weeks, with increased levels of collagen and SOD observed in the CSPE- NLC group.	Lipid nanoparticles	*Citrus sinensis* peel extract	[41]
Different PPEE-SLNs cream formulations (2% and 5%) were assessed for possible anti-wrinkle activity against UV-induced photoaging in a mouse model using a wrinkle scoring method. They demonstrated a highly significant protective effect against UV, as evidenced by tissue biomarkers (SOD) and histopathological studies. The current study demonstrates that *Prunus persica* leaf by-products provide an interesting, valuable resource for natural cosmetic ingredients.	Lipid nanoparticles	*Prunus persica* (L.) leaf extract	[42]
In cellular experiments, A/D-FLip inhibited oxidative stress damage, reduced inflammatory factors and decreased the activation of MMPs in human immortalized keratinocytes (HaCaT) cells. In animal experiments, A/D-FLip inhibited skin damage and reduced skin collagen loss by decreasing the activation of MMPs, thus inhibiting skin photoaging in mice. A/D-FLip has good anti-photoaging effects and it has the potential to become an effective skincare product.	Flexible liposomes	Apigenin and Doxycycline	[43]
The results indicated that liposomes could significantly improve the stability of vitamin D3. Vitamin D3 liposomes repaired the surface morphology of skin in the photoaging model and promoted the production of new collagen fibers. Vitamin D3 liposomes, as a potential skincare agent, significantly improved skin appearance and repair damage in the histology of photoaging.	Liposomes	Vitamin D3	[44]
Spirulina Bilosomes (SPR-BS), a novel anti-aging nanoplatform for SPR delivery, exhibits enhanced in vitro drug release and superior in vivo anti-aging efficacy compared to free SPR	Bilosomes	Spirulina	[45]
Coriander oil possesses anti-wrinkle activity. Coriander oil cream and CEOLNs formulations attenuated in vivo UV-induced skin photoaging, manifested by significantly decreased MDA, COX-2, PGE-2, MMP-1, JNK, and AP-1 levels. These pharmaceutical dosage forms significantly increased skin collagen content compared to the UV-injured group.	Lipid nanoparticle/Nanoemulgel	*Coriandrum sativum* L. essential oil	[46]
The results of the β-galactosidase test showed the promotion of the aging process in the cells treated with the smaller size of ZnO NPs. Both sizes of the NP were found to upregulate the aging-related genes NF-kB and p53 and downregulate the anti-aging gene Nanog. The smaller size of ZnO NPs can enhance the aging process in the cells.	Metallic nanoparticles	Zinc oxide (ZnO)	[47]

**Table 3:** Summary of data collected from clinical studies on new cosmeceuticals delivery systems selected by the exclusion criteria mentioned above.

Results	Delivery System	Active Compound	Reference
Both formulations showed anti-aging potential (anti-wrinkle action, promotion of skin hydration). The emulgel showed greater antioxidant potential.	Emulsion W/O Emulgel	Grape seed extract	[48]
The active formulation recovered the skin from any UV-related imperfections in terms of erythema, melanin, sebum levels, moisture, and elasticity.	Micro emulgel	Flaxseed extract	[49]
It demonstrated effectiveness in treating melasma, surpassing conventional chemical peeling treatment, without causing side effects.	Aspasomes	Ascorbic acid derivative	[50]
The formulation provided clinical improvement of UV-induced damaged skin and ultrastructure, suggesting that spanlastic nanovesicles had a synergistic effect on collagen synthesis and melanin formation in the skin.	Spanlastic vesicles	L-Ascorbic acid	[51]
The formulation demonstrated efficacy at the topical level, offering benefits when applied daily during the two-week trial duration. The formulation also provided visually observable anti-wrinkle effects on the skin, with visible improvements in the skin’s shape and texture.	Micro emulsion	Ginkgo biloba extract	[52]
The adenosine delivery system exhibited enhanced wrinkle improvement of 203% compared to 0.04 wt% of the pure adenosine system. The niacinamide- and sulforaphane-loaded Au-decorated zeolite nanocomposites decreased the skin surface melanin content by 123% and 222%, respectively, compared to 2 and 0.01 wt% of pure niacinamide and sulforaphane systems, respectively.	Metallic nanoparticles	Niacinamide, sulforaphane, and adenosine	[53]
The cream tested on volunteers increased skin hydration, elasticity and had a softening effect. Regarding wrinkles, the cream reduced both their area and depth.	Levan Nanoparticles and Nanoemulsion	Levan from microbial fermentation with *Bacillus subtilis* natto KB1	[54]
The developed polymeric nanoparticles were effective in alleviating wrinkles and can find applications in pharmaceutical formulations utilizing propolis for its antiseptic, anti-inflammatory, antimycotic, antifungal, antibacterial, antiulcer, anticancer, and immunomodulatory properties.	Polymeric nanoparticles	Propolis extract	[55]
A decrease in skin roughness and wrinkle breadth, along with an improvement in dermal homogeneity and firmness, were observed after two months of treatment with “anti-aging” masks. A significant improvement in skin firmness and elasticity was observed after one month of treatment with “lifting” masks. Furthermore, a one-month treatment with “cell renewal” masks promoted the production of new skin cells through a mild exfoliating action.	Bacterial cellulose	Anti-aging mask * Lifting mask * Purifying and regenerative mask *	[56]
The surface evaluation of living skin index values significantly reduced for the emulgels loaded with kaki fruit extract, indicating a reduction in skin wrinkles, roughness and scaliness.	Emulgels	Kaki fruit extract	[57]

* Active ingredients used in the formulation of each type of mask: Anti-aging mask: *Adansonia digitata* fruit extract, *Hibiscus sabdariffa* flower extract, acetyl decapeptide-3, *Coffea arabica* seed cake extract, hydrolyzed hyaluronic acid, palmitoyl tripeptide-38, *Kigelia africana* fruit extract, *Acacia senegal* gum, dimethylaminoethanol tartrate, and *Crocus chrysanthus* bulb extract. Lifting mask: hydrolyzed *Chenopodium quinoa* seed, hydrolyzed hyaluronic acid, and acetyl decapeptide-3. Purifying and regenerative mask: *Portulaca oleracea* flower/leaf/stem extract, sodium hyaluronate cross-polymer, *Chlorella vulgaris* extract, kaolin, and magnesium sulfate.

**Table 4:** Summaries of data collected about cosmeceutical delivery system.

Types of Systems	Carrier Types	Reference
Polymeric-based nanosystems	Polycaprolactone nanofibrous scaffold	[21]
	Polymeric nanoparticles	[55]
Lipid-based nanosystems	Emulgel with niosome	[22]
	Liposomes	[23,24,26,29,31,43,44]
	Nanostructured lipid carriers	[24]
	Nanoemulsion	[24,34,36]
	Niosome	[20,24,30]
	Solid lipid nanoparticles	[27]
	Ethosomes	[31]
	Aspasomes	[50]
	Transethosomes	[30]
	Nano-carriers	[35]
	Hialurosomes	[40]
	Lipid nanoparticles	[41]
	Bilosomes	[45]
	Spanlastic vesicles	[51]
Metal-based nanosystems	Metallic nanoparticles	[32,33,47,53]
Additional systems	Self-emulsifying	[25]
	Encapsulated microparticles	[28]
	Emulgel	[37,57]
	Micelles	[38,39]
	Nanoemulgel	[46]
	Emulsion W/O	[48]
	Micro emulgel	[49,52]
	Levan Nanoparticles	[54]
	Bacterial cellulose	[56]

**Table 5:** Limitations of in vitro studies, system stability, and delivery system ingredients.

Article	Study Limitation	Stability	Delivery System Ingredients
[20]	Although the study reports the anti-aging activity on the skin, it does not delve into the mechanisms by which gallic acid exerts its effects when administered through niosomes.	3 months	Polyoxyethylene 2 cetyl ether, cholesterol, cetyltrimethylammonium bromide
[21]	The experiments focus on short-term exposure to UV stress and the immediate effects of pre-treatment with nanoPcl-M. Long-term effects and the potential for cumulative damage over time are not addressed.	7 days	Polycaprolactone nanofibrous scaffold (PCL), chloroform, ethanol
[22]	The study does not include long-term efficacy data regarding the anti-aging effects of the formulations. The article acknowledges financial support from a cosmetics company.	Not provided	Glycerin dibehenate, cetyl alcohol, cetearyl alcohol, squalene, L-α-phosphatidyl choline from soybean, dimethyl amino ethyl (DMAE), isopropyl palmitate, Tween^®^ 80, Span^®^ 60, cholesterol, 2-phenoxy ethanol, ascorbyl palmitate, disodium EDTA, citric acid, α-tocopherol
[23]	The experiments were conducted over relatively short incubation periods (e.g., 48 to 72 h). The long-term effects of CRPP on cell viability and gene expression were not evaluated.	8 weeks	Cyclodextrins, propanediol, lecithin, tocopherol, sunflower seed oil, Hydrolite^®^ 5 green (pentylene glycol), disodium EDTA
[24]	The study uses a single marker of rosmarinic acid (RA) for the quantitative determination of the extract. Although the study reports zeta potential values and formulation stability, it does not explore the long-term stability of these systems under various environmental conditions.	8 days	Cetyl palmitate, tea seed oil, Tween^®^ 20 (polysorbate 20), Plantacare 2000^®^ (Decyl glucoside), distilled water (DI water), glycerin, propylene glycol, cholesterol
[25]	The study measured only a limited number of cytokines (TNF-α, IL-6, and IL-8) to assess pro-inflammatory activity.	10 months at 5 °C	Fucan-coated acetylated cashew gum nanoparticles, glutaraldehyde, dimethyl sulfoxide (DMSO), lipopolysaccharides (LPS)
[26]	One of the main limitations highlighted is the requirement for royal jelly to be stored at low temperatures and in the absence of light to maintain its stability.	6 months at 6 °C	10-hydroxy-2-decenoic Acid (10-HDA), cyclodextrins, phenolic compounds
[27]	The study does not mention the sample size or the number of independent experiments conducted.	30 days	Precirol^®^ 5 ATO (glyceryl distearate), Gelucire^®^ 39/01, cetyl palmitate, Suppocire DM pellets, Compritol^®^ HD5 ATO, Gelucire^®^ 50/13 (stearoyl polyoxyl-32 glycerides), Suppocire NA15 Pellets, Gelucire^®^ 43/01 (hard fat compounds), Apifil^®^ (PEG-8 beeswax), Dynasan^®^ 114 (glyceryl tristearate), Softisan^®^ 100 (hydrogenated coco-glycerides), Softisan^®^ 154 (hydrogenated palm oil), Witepsol^®^ E76 (hard fat compounds), glyceryl monostearate stearic acid, Pluronic^®^ F-127 (a surfactant), pure anhydrous ethanol, HEPES hemisodium salt, 2,2-diphenyl-1-picrylhydrazyl, DPPH0 uranyl acetate
[28]	The study encountered difficulties in quantifying ascorbic acid (AA) during ex vivo permeation experiments, as the microparticles released AA gradually, resulting in amounts that fell below the detection limits of the analytical method.	Not provided	Acetic acid, trifluoroacetic acid, anhydrous monobasic sodium phosphate, anhydrous bibasic sodium phosphate
[29]	The study does not explore the long-term effects nor the efficacy of liposomal morin in other applications.	Not provided	Lecinolws-50 (Lysolecithin), Tween^®^-80, Pluronic F-68
[30]	The characterization of niosomes focused on size, zeta potential, morphology, and encapsulation efficiency. Other important factors, such as the release kinetics in different biological environments and the potential for skin irritation or allergic reactions, were not extensively addressed.	3 months at −4 °C	Sorbitan monostearate (Span^®^ 40), polyoxyethylenesorbitan monopalmitate (Tween^®^ 40), cholesterol
[31]	Although the study suggests that cycloastragenol (CA) may penetrate the skin barrier when encapsulated in phospholipid vesicles, direct measurement of this penetration remains unconfirmed. The study does not provide long-term data on the efficacy and safety of CA when used in topical applications.	Not provided	Phospholipon^®^ 20H, Sunlipon^®^ 90, Neobee^®^ M-5 oil, xanthan gum, potassium sorbate, glyceryl monostearate (GMS), Alphadim 90 SB, glyceryl monostearate (GMS), Alphadim 90 SB
[32]	The study does not address the long-term effects of GA–AuNPs on skin health and aging.	Not provided	Span^®^ 40 (sorbitan monostearate), Tween^®^ 40 (polyoxyethylenesorbitan monopalmitate), cholesterol, isopropyl alcohol, chloroform and acetone, sodium borate buffer solution (pH 9.1)
[33]	The study does not include long-term exposure assessments, which are crucial for understanding the chronic effects and potential cumulative toxicity of AuNP over time.	25 days	Chloroauric acid, sodium citrate, citrate
[34]	The article does not provide comprehensive data on the long-term stability of nanoemulsions under various environmental conditions (e.g., light exposure, humidity).	30 days	Coco-betaine, surfactin, carbon dioxide
[35]	The study evaluates the immediate effects on cell viability and gene expression but does not address the long-term impacts of these nanocarriers on skin repair and aging processes.	Not provided	Mineral, sesame, and soybean oils, ethanol, nanocarrier base
[36]	The effects of prolonged storage or exposure to different environmental conditions on the stability of IB@NPs were not evaluated.	28 days	Idebenone (IB), polyethylene glycol-40 hydrogenated castor oil (PEG-40 HCO), deionized water (DIW)

**Table 6:** Limitations of in vivo studies, system stability, and delivery system ingredients.

Article	Study Limitation	Stability	Delivery System Ingredients
[37]	Data on the drug release profile and its dermal penetration are missing. The ages of the mice used in the anti-aging activity and skin irritation tests were not specified.	28 days	Coenzyme Q10 (CoQ10) L-α-Phosphatidylcholine oleic acid, Tween^®^ 80, Span^®^ 80, Carbopol 940, triethanolamine (TEA)
[38]	The study used 5-week-old mice to assess epidermal proliferation and 8-week-old male guinea pigs to investigate pharmacological effects. However, the number of animals used in the experiment was not reported. Alpha-lipoic acid was applied as an antioxidant supplement for anti-aging purposes, but the study did not mention the aging factor of the animals. In addition, the study did not report any stability testing.	Not conducted	Alpha-lipoic acid (α-LA), non-ionic surfactant (BS-20), magnesium chloride (MgCl_2_), sodium bicarbonate (NaCO), sodium hydroxide solution (NaOH) HEPES
[39]	The article highlights the physical instability of emulsions with L-ascorbic acid, despite no perceptible degradation of the ingredient.	45 days	FucoPol L-ascorbic acid (vitamin C), α-tocopherol (vitamin E), olive oil (Olea europaea), sweet almond oil (Prunus amygdalus dulcis), cetyl alcohol, glycerin methylparaben triethanolamine (TEA)
[40]	The experiment was conducted on the shaved dorsal skin of adult male Wistar rats, aged between 6 and 8 weeks.	3 months	Caffeine (CAF), phospholipids (Phospholipon^®^ 90G), hyaluronic acid (hyaluronan), Carbopol 940
[41]	The study does not present more robust stability tests, such as long-term tests under different temperature and humidity conditions, and it also does not address the potential sensitivity of human skin to the formulation or allergic reactions.	Not provided	Sweet orange peel extract (citrus sinensis), lipid nanoparticles (LNPs), cocoa butter, olive oil, Tween^®^-80, phosphatidylcholine (Lecithin)
[42]	The mice used in the tests were young—only 4 weeks old.	2 months	Peach leaf extract (PPEE), solid lipid nanoparticles (SLNs): glyceryl monostearate, Tween^®^ 80. Cream base ingredients: Span^®^ 80, Cetearate-20, liquid paraffin, cetostearyl alcohol, beeswax biocrol WS2, propylene glycol, purified water
[43]	The age of the mice was not provided.	28 days	Apigenin (98% purity), doxycycline (98% purity), lecithin, cholesterol, sodium cholate, Tween^®^ 80, vitamin E, phosphate buffer saline (PBS)
[44]	The age of the rats was not provided.	9 days	Egg phosphatidylcholine (Egg PC), phosphate buffer saline (PBS), phospholipids, cholesterol, vitamin D_3_, ethanol
[45]	The experiment was conducted on the shaved dorsal skin of adult male Wistar rats, aged between 6 and 8 weeks.	3 months	Sodium deoxycholate (SDC), phospholipids, phosphate, buffer saline (PBS), spirulina (SPR), lipoid S100 (l-α-PC), cholesterol
[46]	The mechanisms underlying the anti-aging properties of coriander oil have not yet been fully characterized. Furthermore, the stability tests are not clearly described in the methodology. Eight-week-old female mice were used in the tests, where photoaging was induced by UV radiation, and erythema formation was assessed 24 h after exposure.	Not provided	Coriander essential oil-loaded solid lipid nanoparticle (COEOLNs): coriander essential oil, cocoa butter, olive oil or sesame oil and lecithin; nanoemulgel with CEONLIC: xanthan gum, deionized water, Tween^®^ 80, triethanolamine.
[47]	The study highlights a size-dependent toxicity of nanoparticles (NPs); however, it does not delve deeper into the underlying mechanisms of this phenomenon. Furthermore, the study does not report on the stability of the system.	Not provided	Zinc oxide (ZnO), phosphate buffer saline (PBS)

**Table 7:** Limitations of clinical studies, system stability, and delivery system ingredients.

Article	Study Limitation	Stability	Delivery System Ingredients
[48]	The tested formulation included potentially harmful ingredients, such as parabens. The study involved 40 individuals, all classified as healthy. Furthermore, the lack of demographic data, such as the participants’ ages, is a relevant limitation.	90 days	Emulsion W/O: propylene, paraben, paraffinoil, Abil-EM, methylparaben, olive oil Emulgel: Emulsion ingredients + Carbapol 940, triethanolamine
[49]	The study included 14 healthy Asian women, with the participants’ age range varying only between 25 and 40 years. Another limitation to consider is that this is a monocentric and single-blind study.	90 days at 25 °C	Tween^®^ 80, Span^®^ 80, ethanol, isopropyl myristate
[50]	The study included only 20 Egyptian women, with an age range of 25 to 60 years. Additionally, it is a monocentric, single-blind study. A positive aspect is that the participants had melasma, rather than being only healthy individuals.	3 months	Ascorbyl palmitate, lecithin, cholesterol, magnesium ascorbyl phosphate, phosphate buffered saline
[51]	Although the study was evaluated both in vitro and in vivo, the research involving humans was quite limited, as it included only 6 healthy female volunteers, all with normal skin condition.	6 months	Span^®^ (40, 60, or 80), tween^®^ (60 or 80) or tocopherol polyethylene glycol 1000 succinate, ethanol
[52]	The formulation contains parabens. The study included only 12 participants of both sexes, conducted at a single center in Egypt.	1 month, both 4 and 25 °C	Stearic acid, potassium hydroxide, methylparaben, glycerol
[53]	The study presents significant limitations, such as the lack of an exact number of participants, indicating only that there were more than 20 women. Additionally, no information was provided about the sex and age range, mentioning only that all were over 19 years old. The conclusion is quite general.	Not provided	Calcined zeolite 13X, gold chloride hydrate, oleic acid
[54]	The study had a small sample size, with only 10 women aged between 42 and 54 years.	90 days	Nanoemulsion: sodium surfactin powder, 2-(2 ethoxyethoxy) ethanol, ascorbyl tetraisopalmitate
[55]	In this study, no significant limitations were identified. It is a double-blind study with an adequate number of volunteers, covering an appropriate age range.	6 months at 25 ± 2 °C, 3 months at 40 ± 2 °C	PN7-PEG 400; Poloxamer 188; Poloxamer 407
[56]	The study included about 20 to 25 volunteers for each type of application. Although the sample size is relatively small, there was careful consideration of the age range of the participants: the anti-aging mask was tested on individuals aged between 42 and 64 years, the lifting mask on people aged between 38 and 57 years, and the purifying and regenerative mask on participants aged between 25 and 40 years.	Not provided	Bacterial Cellulose
[57]	The sample consisted of 13 healthy men of Asian origin, aged between 25 and 37 years. The study used a single-blind method. Additionally, the formulation included parabens, a potentially harmful ingredient.	Not provided	Span^®^ 80, Tween^®^ 80, methylparaben, water, Carbopol-940

## Data Availability

Data are contained within the article.
